# Radiosensitive HSPC Subsets Define Early Hematopoietic Injury and Enable H-ARS Therapeutic Screening

**DOI:** 10.3390/cimb48080801

**Published:** 2026-08-07

**Authors:** Hyun Bo Sim, Dae-Han Park, Seul-Ki Mun, Yu-Jeong Choi, Ho Seong Seo, Seung-Hyun Jeong, Dong-Jo Chang, Jong-Jin Kim

**Affiliations:** 1 Department of Biomedical Science, Sunchon National University, 255 Jungang-ro, Suncheon-si 57922, Republic of Korea; kokonun3@naver.com (H.B.S.); carbdsinc@naver.com (D.-H.P.); motomoto1210@naver.com (S.-K.M.); pskd357@naver.com (Y.-J.C.); 2 R&D Team, Suncheon Research Center for Bio Health Care, Suncheon 57962, Republic of Korea; 3 Korea Radio-Isotope Center for Pharmaceuticals, Korea Institute of Radiological & Medical Sciences, Seoul 01812, Republic of Korea; 4 Research Division for Cyclotron Application, Korea Atomic Energy Research Institute, Jeongeup 56212, Republic of Korea; hoseongseo@kaeri.re.kr; 5 Research Institute of Life and Pharmaceutical Sciences, College of Pharmacy, Sunchon National University, Suncheon-si 57922, Republic of Korea; jeongsh@scnu.ac.kr

**Keywords:** H-ARS, HSPCs, CyTOF, radiosensitive progenitors, therapeutic screening

## Abstract

Early hematopoietic injury is a critical determinant of hematopoietic acute radiation syndrome (H-ARS), yet biologically relevant cellular endpoints and optimal evaluation windows for therapeutic screening remain poorly defined. Here, we combined high-dimensional mass cytometry (CyTOF) and flow cytometry to characterize early hematopoietic stem and progenitor cell (HSPC) remodeling following 6.5 Gy total-body irradiation. Radiation exposure induced rapid bone marrow injury, with substantial cellular loss and reduced viability occurring within hours after irradiation. CyTOF analysis revealed that radiation-induced injury was characterized by selective remodeling rather than uniform depletion of the HSPC compartment. While long-term hematopoietic stem cells (LT-HSCs) were relatively preserved, short-term HSCs (ST-HSCs), multipotent progenitors (MPPs), and megakaryocyte–erythroid progenitors (MEPs) exhibited marked reductions during the early phase after irradiation. Importantly, surviving cells retained partial differentiation capacity during this period, indicating that the early post-irradiation phase represents a biologically informative window for therapeutic evaluation. These radiosensitive populations were subsequently validated using a simplified flow cytometry platform and remained detectable under short-term in vitro culture conditions. Collectively, our findings identify key radiosensitive HSPC subsets and establish an early hematopoietic injury framework that integrates optimal evaluation timing with practical cellular endpoints for H-ARS therapeutic screening and radiomitigator development.

## 1. Introduction

Radiation is widely utilized in energy production, national defense, industrial applications, and medical treatments. However, accidental or intentional exposure to high doses of ionizing radiation can cause severe biological damage and result in acute radiation syndrome (ARS) [1,2,3]. Depending on the absorbed dose and exposure duration, ARS is classified into hematopoietic (H-ARS), gastrointestinal (GI-ARS), and cerebrovascular (CNS-ARS) syndromes [4,5]. Among these, H-ARS is the most common form and is characterized by rapid depletion of bone marrow (BM) cells, resulting in neutropenia, lymphopenia, thrombocytopenia, and increased susceptibility to infection and sepsis [6,7]. In addition to acute mortality, survivors may develop delayed effects of acute radiation exposure (DEARE), which contribute to long-term multi-organ dysfunction and reduced quality of life [8,9].

The hematopoietic system is particularly vulnerable to radiation-induced injury because hematopoietic stem and progenitor cells (HSPCs) continuously maintain blood and immune cell production throughout life. Radiation exposure induces DNA double-strand breaks (DSBs), oxidative stress, and apoptotic signaling, ultimately disrupting hematopoietic homeostasis [10,11,12]. In mice, HSPCs are broadly classified into Lineage-negative (Lin-) populations, including long-term hematopoietic stem cells (LT-HSCs), short-term HSCs (ST-HSCs), multipotent progenitors (MPPs), common myeloid progenitors (CMPs), common lymphoid progenitors (CLPs), and downstream progenitor populations [13,14,15,16]. Because these populations represent the cellular foundation of hematopoietic regeneration, their preservation and recovery are critical determinants of survival following radiation exposure [17].

Several medical countermeasures (MCMs) have been approved for H-ARS treatment, including granulocyte colony-stimulating factor (G-CSF), granulocyte–macrophage colony-stimulating factor (GM-CSF), and the thrombopoietin receptor agonist romiplostim [18,19,20,21,22]. Although these agents improve hematopoietic recovery, their efficacy remains incomplete and clinical limitations still exist [5,18]. Consequently, the development of novel radioprotective and radiomitigative compounds remains an important priority for both public health preparedness and radiation medicine.

A major challenge in H-ARS therapeutic development is the lack of biologically informative early endpoints that can be used for rapid compound evaluation. Most preclinical studies assess therapeutic efficacy using survival, peripheral blood recovery, or bone marrow regeneration at relatively late time points after irradiation. While these endpoints are clinically relevant, they provide limited insight into the earliest cellular events that determine subsequent hematopoietic collapse or recovery. Furthermore, systematic analyses of HSPC dynamics during the first 24 h following radiation exposure remain limited, leaving the earliest stages of hematopoietic injury incompletely characterized. Identification of radiosensitive HSPC populations during this critical period could facilitate the development of quantitative screening strategies and improve the efficiency of H-ARS therapeutic discovery.

In the present study, we investigated the early remodeling of HSPC populations following 6.5 Gy total-body irradiation using high-dimensional mass cytometry (CyTOF). We first characterized temporal changes in HSPC populations during the early phase of radiation injury and identified radiosensitive progenitor subsets associated with hematopoietic collapse. These findings were subsequently translated into a simplified flow cytometry-based platform and further evaluated under short-term in vitro culture conditions. Together, our study provides a biologically informed framework for monitoring early hematopoietic injury and establishes practical cellular endpoints for future H-ARS therapeutic screening and radiomitigator development.

## 2. Materials and Methods

### 2.1. Animal Information

This animal experiment was approved by Sunchon National University Institutional Animal Care and Use Committee (SCNU IACUC-2025-14). C57BL/6 mice (male, 9~12 weeks, NCrlOr) were purchased from Orient Bio (Seongnam, Republic of Korea). The animals were housed in polycarbonate cages in a constant-temperature, constant-humidity environment (22 ± 2 °C, 50 ± 5% humidity) and provided with standard feed and water.

### 2.2. Irradiation Assay

Mice received a single dose of total-body irradiation (6.5 Gy) using a 137Cs γ-ray source (Gammacell 40 Exactor, Nordin International Inc., Kanata, ON, Canada) at a dose rate of 0.8 Gy/min, with 10 mice irradiated simultaneously. Irradiation was performed at room temperature. Non-irradiated controls were handled under identical conditions. Mice were sacrificed at the early (4–6 h), middle (24 h), and late (48 h) post-irradiation points.

### 2.3. BM Cells Extraction and Cultures

BM cells were harvested from the tibia and femur using RPMI 1640 medium (Hyclone, Novato, CA, USA) supplemented with 10% fetal bovine serum (FBS, Hyclone, Novato, CA, USA), 2-mercaptoethanol (2-ME, Thermo, Waltham, MA, USA), 25 mM hydroxyethyl piperazine ethane sulfonicacid (HEPES, Thermo, Waltham, MA, USA), 1% sodium pyruvate (Thermo, Waltham, MA, USA), and 1% antibiotic–antimycotic (Thermo, Waltham, MA, USA). Red blood cells (RBCs) were lysed using RBC lysis buffer (Thermo, Waltham, MA, USA). Total cell counts, including percentage of live and dead cells, were determined using an automated cell counter (Countess 3, Thermo, Waltham, MA, USA) with trypan blue staining (Thermo, MA, Waltham, USA). BM cells were cultured at 37 °C in 5% CO_2_.

### 2.4. Viability Assay

BM cells were seeded in triplicate in 96-well plates (5 × 10^5^ cells/well). After incubation, 100 μL of culture medium was removed from each well, and 10 μL of CCK-8 reagent (Cell Counting Kit-8, Dojindo, Kumamoto, Japan) was added. Optical density (OD) was measured using a microplate reader (VersaMax, Molecular Devices, Sunnyvale, CA, USA). Cell viability was calculated as the ratio of OD at 24 h to OD at 0 h.

### 2.5. Antibody Metal Labeling

Metals were conjugated to purified antibodies using Maxpar X8 Antibody Labeling Kits (Standard Biotools, San Francisco, CA, USA). Experiments were performed according to the manufacturer’s (Standard Biotools) instructions. Of the 19 antibodies used in the experiment, 3 (CD172α/SIRPα-155Gd, CD134/Flt-3-173Yb, and CD34-176Yb) were conjugated to each metal.

### 2.6. Mass Cytometry Analysis

Harvested mouse spleens were prepared into single-cell suspensions, with 1 × 10^7^ cells for each sample. Cells were stained using a panel of metal-conjugated antibodies (Table 1) after cisplatin (Cell-ID Cisplatin, Standard Biotools, San Francisco, CA, USA) live/dead staining. DNA was labeled with an Ir-intercalator (Cell-ID Intercalator-Rh, Standard Biotools, San Francisco, CA, USA) to detect the presence of cells. EQ Bead (EQ Four Element Calibration Beads; Standard Biotools, San Francisco, CA, USA) was added to each sample immediately before analysis. The study was performed using the Helios (CyTOF2, Standard Biotools, San Francisco, CA, USA), and 300,000 events were collected for each sample [23,24]. All fcs files were analyzed using Cytobank (v10.6, Beckman Coulter, https://premium.cytobank.org/cytobank/experiments (accessed on 14 June 2026)).

### 2.7. Dimensionality Reduction Analysis

t-SNE (t-distributed stochastic neighbor embedding) analysis was used to express the data after the clean-up process by dimension reduction as a 2D map. Map generation was performed using the opt-SNE (optimized t-SNE) algorithm, and the analysis was based on bone marrow cells (hematopoietic stem cells and precursor cells, lineage-out population), with a total proportional-event sampling of 30,000.

### 2.8. FlowSOM Analysis

All clustering analyses were performed based on the previously obtained t-SNE map. FlowSOM (self-organizing map) analysis was performed, and 100 clusters were formed using the Hierarchical Consensus clustering method to get 10 metaclusters. At this time, the seed value was randomly optimized. The ten metaclusters were named into 13 types of cells (LT-HSC, CMP, GMP, CMP, MEP, ST-HSC, MPP01/02, CLP01/02/03, MDP, CDP) through the difference in the expression rate of 8 signature markers (Sca-1, c-Kit, CSF-1R, CD16/32, CD150, Flt-3, IL-7Rα, CD34). The data obtained through analysis were visualized and graphed using SRplot (A free online platform for data visualization and graphing).

### 2.9. BM Cell Stimulation and Crystal Violet Stain

BM cells were seeded in triplicate in 24-well plates (5 × 10^5^ cells/well) and stimulated with 1000 U/mL GM-CSF for 5 days. Cells were fixed and stained with crystal violet. Images were captured using an EVOS M7000 imaging system (Thermo, Waltham, MA, USA) with a 10× objective.

### 2.10. Differentiation Analysis

BM cells were seeded in triplicate in 24-well plates (5 × 10^5^ cells/well) and stimulated with 1000 U/mL GM-CSF for 5 days. Cells were blocked with FCγR using anti-CD16/32 antibody (2.4G2, BD Biosciences, Franklin Lakes, NJ, USA), then stained with PE-Ly6G/C (RB6-8C5, BioLegend, San Diego, CA, USA) and APC-Cy7-F4/80 (QA17A29, BioLegend, San Diego, CA, USA). The Ly6G/C^+^ or F4/80^+^ cell populations were analyzed by flow cytometry (FACS Canto II system, BD Biosciences, Franklin Lakes, NJ, USA). Data was analyzed with FlowJo software version 10.10.0 (TreeStar, Ashland, OR, USA).

### 2.11. Flow Cytometry Assay (HSPC Analysis)

BM cells (2 × 10^6^ cells) were seeded in 96-well plates. Cells were blocked with FCγRusing anti-CD16/32 antibody (2.4G2, BD Biosciences, NJ, USA)and stained with BV421-CD150 (TC15-12F12.2, BioLegend, San Diego, CA, USA), BV-605-Sca-1 (D7, BioLegend, San Diego, CA, USA), and BV711 (A7R34, BioLegend, San Diego, CA, USA) in Brilliant Stain Buffer (BD Bioscience, Franklin Lakes, NJ, USA). Cells were washed and stained with Alexa Fluor 488-Lineage (CD3ε, CD11b, CD11c, Gr-1, NK1.1, and CD19), PE-Flt-3 (A2F10; BioLegend, San Diego, CA, USA), PE-Cy5-CD34 (MEC14.7; BioLegend, San Diego, CA, USA), and PE-Cy7-c-kit (ACK2; BioLegend, San Diego, CA, USA). HSPC populations were measured by flow cytometry (FACS Canto II system, BD Biosciences, Franklin Lakes, NJ, USA) and analyzed using FlowJo software version 10.10.0 (TreeStar, Ashland, OR, USA). To visualize changes in Lin^−^ Sca-1^low^ c-Kit^low^ populations, t-SNE maps were generated using the t-SNE plug-in provided in FlowJo software.

### 2.12. Statistical Analysis

Statistical analysis was performed using Student’s *t*-test for comparison between two groups, and One-way analysis of variance (ANOVA) followed by Tukey’s HSD for comparison between more than two groups. Data analyses were conducted using SPSS v29 (SPSS, Chicago, IL, USA). After ANOVA, a post hoc test was conducted only when the significance between groups was *p* < 0.05. Symbols (* *p* < 0.05, ** *p* < 0.01, *** *p* < 0.001) indicated subsequent comparisons of significance between groups. Data are presented as mean ± standard deviation (SD). Significance comparisons were presented based on the Control group.

## 3. Results

### 3.1. Early Hematopoietic Injury Develops Rapidly Following Radiation Exposure

**Figure 1:**
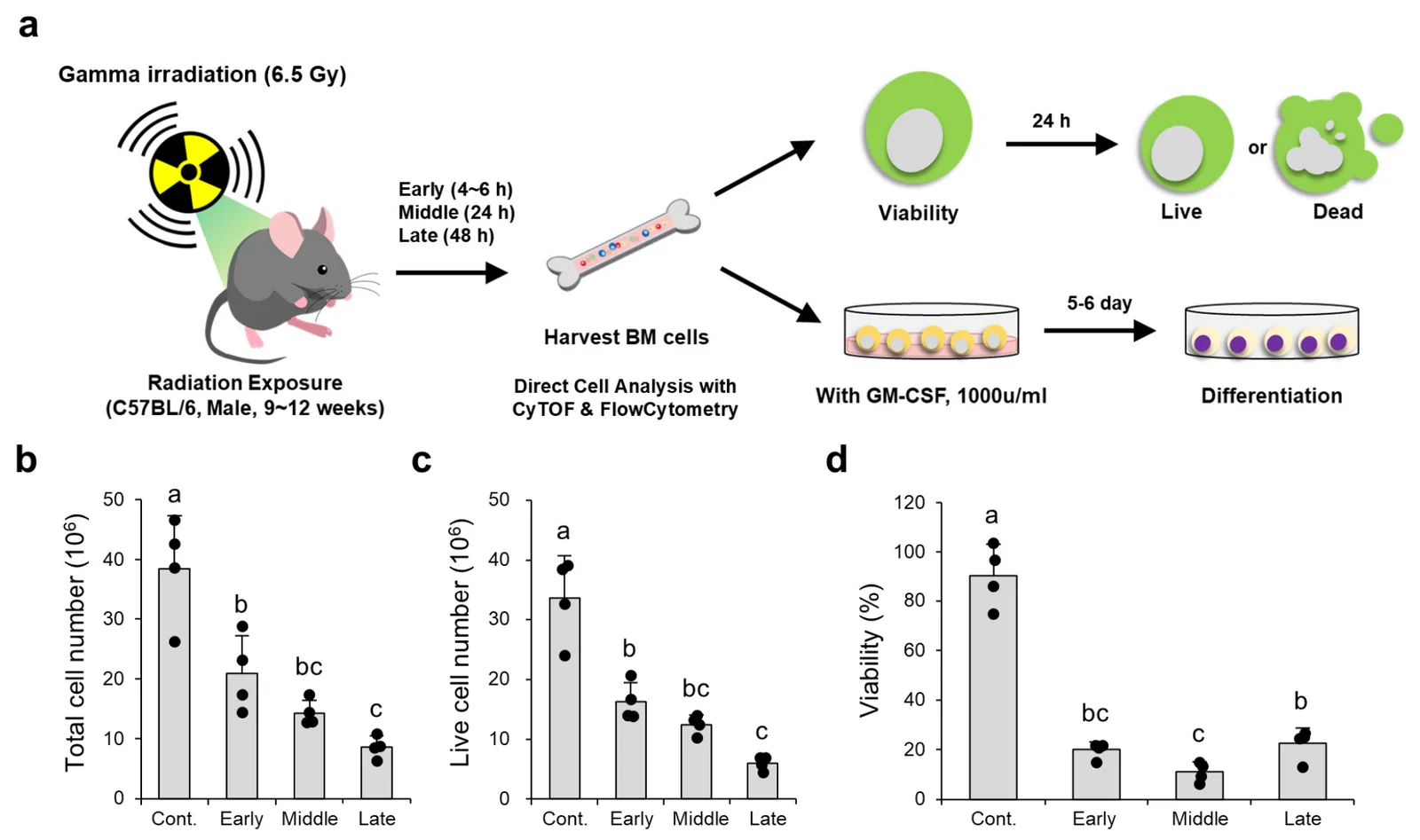
Damage to BM cells due to radiation exposure. (**a**) Experimental scheme of BM from the radiation exposure of C57BL/6 mice (9~12 weeks). Mice were divided into four groups (Cont., Early, Middle, Late, *n* = 5). (**b**,**c**) The numbers of total cells and live cells in the BM were quantified after the mice were sacrificed at each time point using an automated cell counter. (**d**) Viability of BM (5 × 10^5^ cells) was measured 24 h post-harvest using CCK-8 assay (*n* = 4). Data was presented as mean ± SD. The reported *p*-values were obtained from a one-way analysis of variance (ANOVA). Different letters (a–c) indicate significant differences.

Mice were sacrificed at three points (Early, Middle, and Late) following 6.5 Gy total-body irradiation (TBI) to characterize the temporal progression of radiation-induced bone marrow injury (Figure 1a). The total number of BM cells (live + dead) progressively decreased after irradiation, with reductions of 46%, 63%, and 78% observed at the Early, Middle, and Late time points, respectively, compared with non-irradiated controls (Figure 1b). Live BM cell numbers showed a similar decreasing pattern (Figure 1c). To evaluate radiation-induced functional impairment, BM cells were cultured for 24 h, and viability was assessed. While non-irradiated BM cells maintained approximately 90% viability, irradiated BM cells exhibited a marked reduction to below 25% viability at the Early time point, with similarly low levels observed at the Middle and Late time points (Figure 1d). These results demonstrate that substantial bone marrow injury occurs within hours following irradiation and indicate that critical hematopoietic changes emerge during the early phase after radiation exposure.

### 3.2. CyTOF Reveals Hierarchical Remodeling of HSPC Populations Following Radiation Exposure

**Figure 2:**
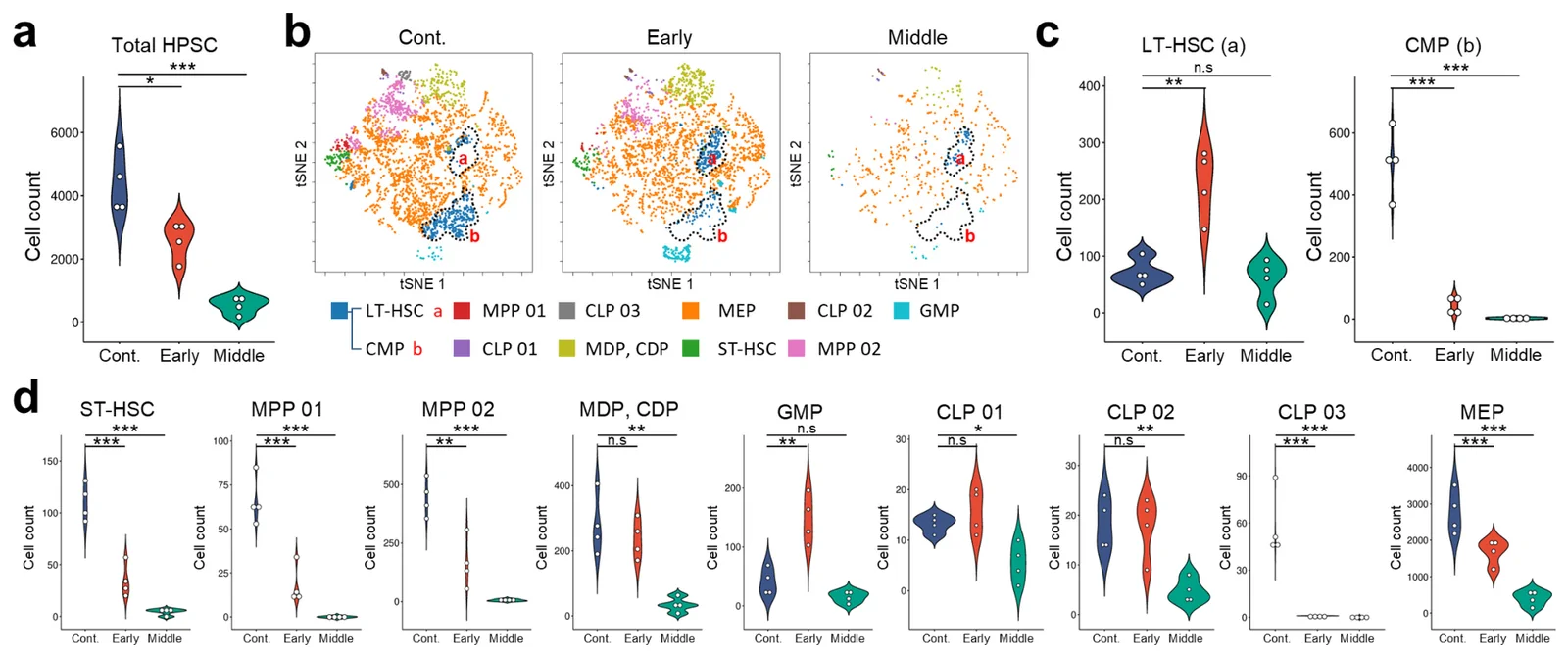
HSPC analysis using the 19-antibody panel of CyTOF. (**a**) Total number of HSPCs (*n* = 4). (**b**) FlowSOM analysis for comparing HSPC changes over time after radiation exposure; a and b (black dotted line) identifying and marking regions within the blue metacluster that exhibit differences over time. (**c**) Changes in cell numbers in a and b of the blue metacluster. (**d**) Changes in cell numbers over time within 9 metaclusters. The reported *p*-values were obtained from a one-way analysis of variance (ANOVA) and correspond to two-sided statistical tests. (* *p* < 0.05, ** *p* < 0.01, *** *p* < 0.001; n.s: not significant).

To characterize radiation-induced alterations within the hematopoietic stem and progenitor cell (HSPC) compartment, CyTOF analysis was performed using a 19-marker panel. Following lineage exclusion, ten major HSPC populations were identified and quantified (Figure S1a–c). We subsequently compared HSPC dynamics between the Early and Middle phases following irradiation. Consistent with the overall reduction in bone marrow cellularity, the total number of HSPCs progressively declined after irradiation, decreasing by 50.6% in the Early group and 87.7% in the Middle group compared with non-irradiated controls (Figure 2a). To further investigate subset-specific responses, FlowSOM clustering was applied to identify radiation-sensitive HSPC populations. Among the identified metaclusters, LT-HSCs and CMPs exhibited markedly different response patterns over time (Figure 2b). Quantitative analysis revealed a transient enrichment of LT-HSCs during the Early phase, followed by a return toward baseline levels at the Middle time point. In contrast, CMPs showed a profound and progressive decline following irradiation (Figure 2c). Analysis of the remaining HSPC populations demonstrated that ST-HSCs, MPP01, MPP02, CLP03, and MEPs were significantly reduced during both the Early and Middle phases (Figure 2d). In contrast, MDPs, CDPs, CLP01, and CLP02 remained relatively stable during the Early phase. Notably, GMPs exhibited a transient increase during the Early phase before declining at the Middle time point (Figure 2d).

Collectively, these findings demonstrate that radiation-induced hematopoietic injury is accompanied by non-uniform remodeling of the HSPC compartment. Rather than causing equivalent depletion of all progenitor populations, radiation exposure generated a hierarchical pattern of cellular vulnerability characterized by relative preservation of LT-HSCs and preferential loss of ST-HSCs, MPPs, MEPs, and CMPs.

### 3.3. Residual Hematopoietic Differentiation Capacity Is Preserved During the Early Phase After Irradiation

**Figure 3:**
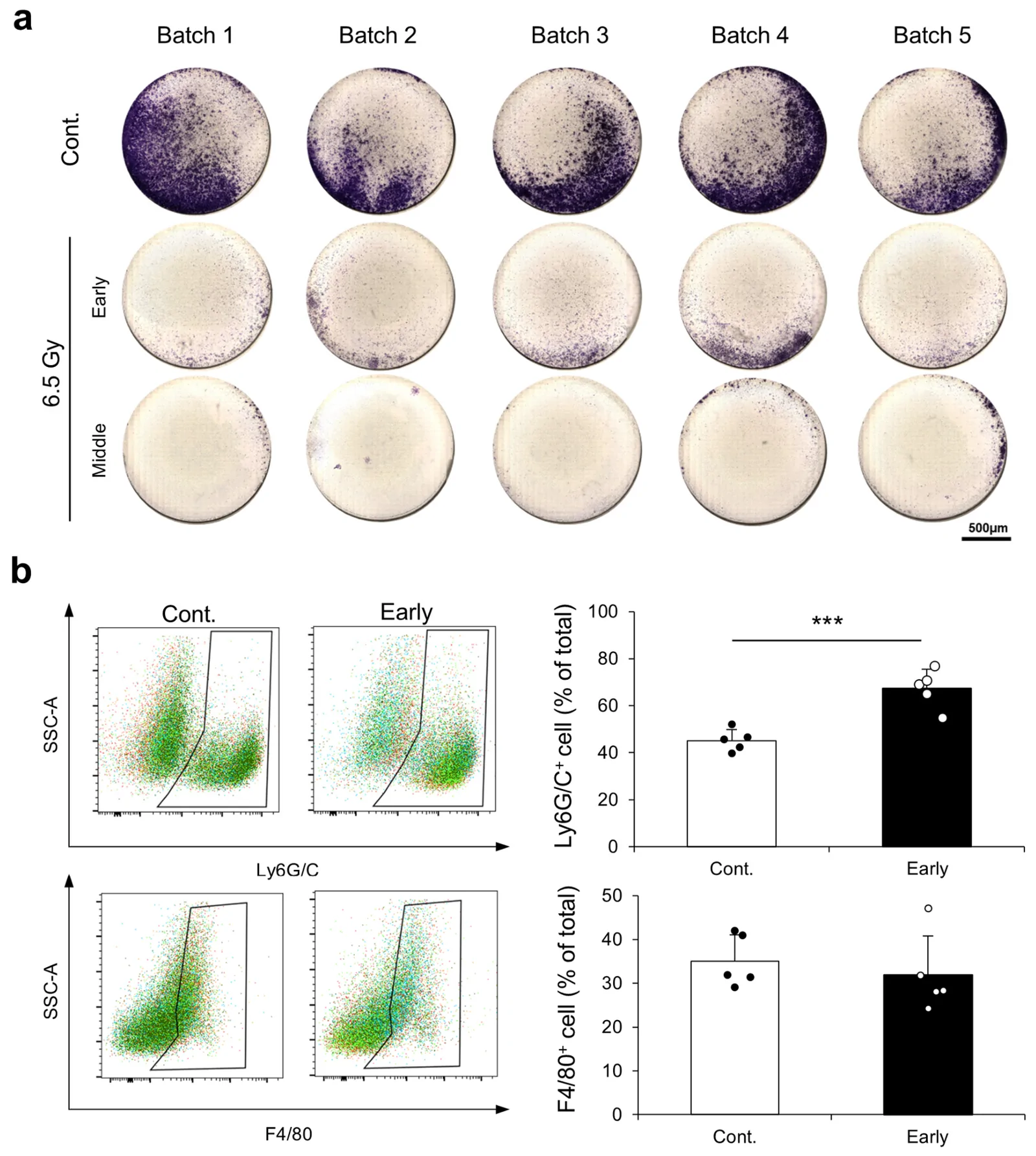
Differentiation of BM after stimulation with GM-CSF. (**a**) BM (5 × 10^5^ cells) was stimulated with 1000 U/mL GM-CSF for 5 days. Differentiation was measured by crystal violet stain and imaged using the EVOS M7000 imaging system (*n* = 5, ×10 air objective). Scale bars: 500 μm. (**b**) A population of Ly6G/C^+^ or F4/80^+^ cells in the differentiation of BM was analyzed by flow cytometry. The different colors in the dot plot represent each batch within the group. Data are presented as mean ± SD. The reported *p*-values were obtained from Student’s *t*-tests. (*** *p* < 0.001).

To determine whether surviving bone marrow cells retained functional potential following irradiation, BM cells collected at the Early and Middle time points were stimulated with GM-CSF and evaluated for colony formation and myeloid differentiation (Figure 3a). Crystal violet staining demonstrated a marked reduction in cell numbers in irradiated groups compared with non-irradiated controls. Colony formation was substantially reduced at the Early time point and was rarely observed at the Middle time point, indicating progressive loss of hematopoietic regenerative capacity following radiation exposure.

Flow cytometric analysis revealed that despite the severe reduction in cell numbers, a subset of surviving BM cells retained the ability to differentiate into macrophages (F4/80^+^) and granulocytes (Ly6G/C^+^) following GM-CSF stimulation (Figure 3b). Notably, the relative frequencies of these differentiated populations were comparable to, or higher than, those observed in non-irradiated controls. These findings suggest that although substantial hematopoietic injury occurs shortly after irradiation, a fraction of surviving cells retains functional differentiation potential during the early phase. In contrast, this capacity was largely diminished by the Middle time point. Collectively, these results indicate that the early post-irradiation phase represents a biologically informative window during which radiation-induced hematopoietic injury can still be functionally evaluated.

### 3.4. Flow Cytometry Validates Radiosensitive HSPC Populations Identified by CyTOF

CyTOF analysis revealed that radiation-induced remodeling of the HSPC compartment was already evident during the Early phase following irradiation. To determine whether these changes could be reproduced using a simplified and more accessible platform, a reduced antibody panel was applied to flow cytometric analysis of freshly isolated bone marrow cells collected at the Early time point.

**Figure 4:**
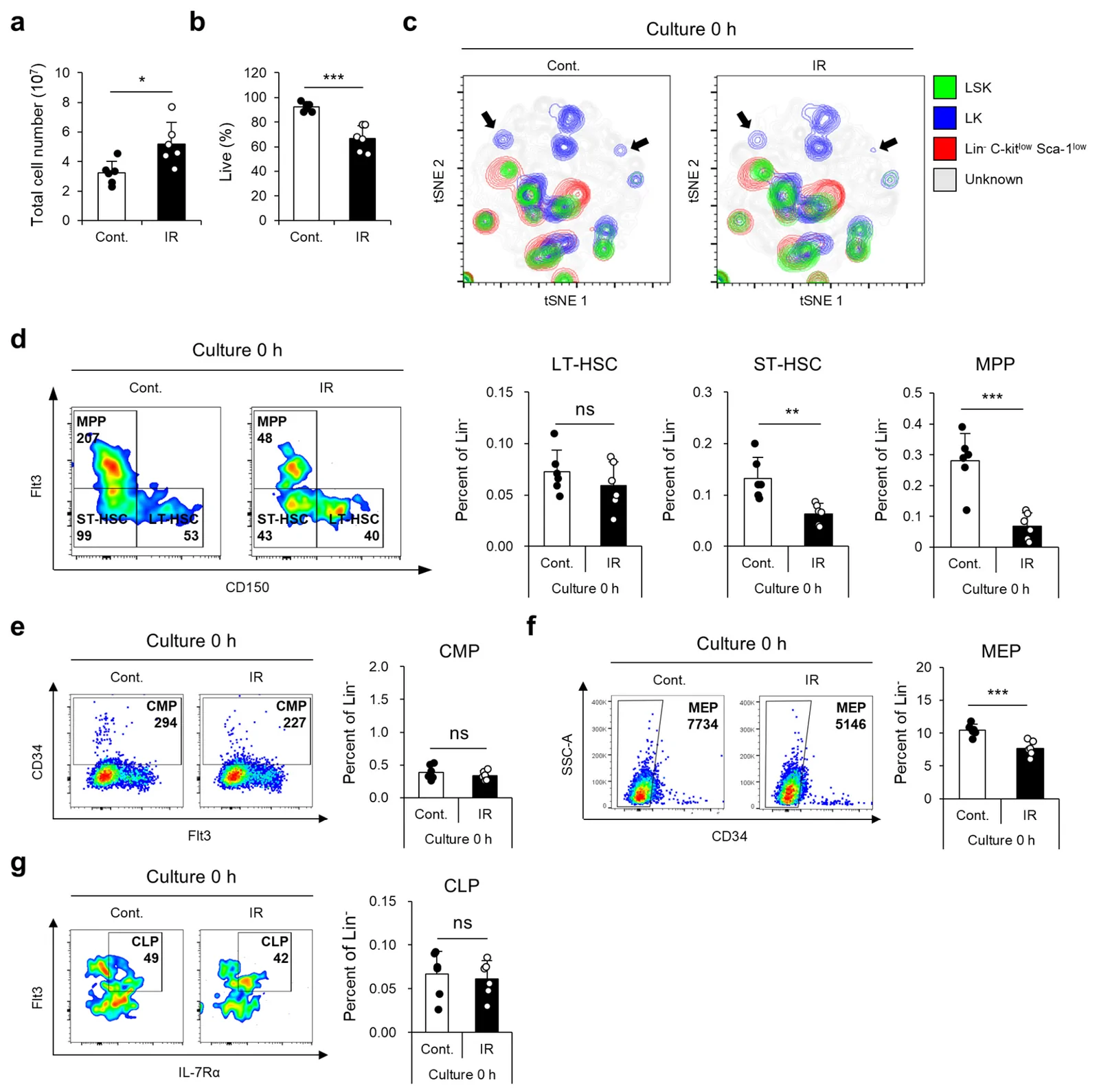
HSPC analysis using the 7-antibody panel. (**a**,**b**) Total cell numbers and percentage live (%) BM cells were measured using a cell counter. (**c**) The populations in LSK (Lin^−^ Sca-1^+^ c-Kit^+^), LK (Lin^−^ Sca-1^−^ c-Kit^+^), and Lin^−^ Sca-1^low^ c-Kit^low^ cells within Lin^−^ cells were visualized using a t-SNE map. The black arrows indicate areas on the tSNE map where there is a large difference between groups. (**d**–**g**) After performing Lin- (anti-CD3ε, anti-Gr-1, anti-CD11c, anti-CD11b, anti-CD19, and anti-NK1.1) gating, only HSPCs (c-Kit, Sca-1) remained and were then gated individually in combination to identify each subpopulation. (**d**) After performing c-Kit^+^ and Sca-1^+^ gating, LT-HSC (Flt3^−^ CD150^+^), ST-HSC (Flt3^−^ CD150^−^), and MPP (Flt3^+^ CD150^−^) were identified using Flt3 and CD150. (**e**) c-Kit^+^, Sca-l^−^, CD34^+^, and Flt3^+/−^ were identified MEP. (**f**) c-Kit^+^, Sca-l^−^, and CD34^−^ were identified MEP. (**g**) c-Kit^low^, Sca-l^low^, IL-7Rα^+^, and Flt3^+^ was identified CLP. The HSPCs population was quantified through flow cytometry. The populations were quantified through flow cytometry. The graph shows the percentage (%) of the LT-HSC, ST-HSC, MPP, CMP, MEP, and CLP. Dot plots present representative images and represent the mean cell number values. Data was presented as mean ± SD. The reported *p*-values were obtained from Student’s *t*-tests. (* *p* < 0.05, ** *p* < 0.01, *** *p* < 0.001, ns: not significant).

Although the total number of recovered BM cells increased following irradiation (Figure 4a), the proportion of live cells was significantly reduced (Figure 4b). Multidimensional flow cytometric analysis demonstrated a decrease in the LK (Lin^−^ c-Kit^+^ Sca-1^−^) population, whereas the frequencies of LSK (Lin^−^ c-Kit^+^ Sca-1^+^) and Lin^−^ Sca-1^low^ c-Kit^low^ populations remained relatively unchanged (Figure 4c). To further characterize radiation-sensitive HSPC populations, six major HSPC subsets were analyzed (Figure S2a). Consistent with the CyTOF results, LT-HSC frequencies were not significantly altered following irradiation (Figure 4d). In contrast, ST-HSCs and MPPs were significantly reduced by 57% and 77%, respectively (Figure 4d). CMPs and CLPs showed no significant changes (Figure 4e,f), whereas MEPs exhibited a significant reduction of 26% compared with non-irradiated controls (Figure 4g).

Taken together, these findings demonstrate that the selective reduction of ST-HSCs, MPPs, and MEPs observed by CyTOF can be reproducibly detected using conventional flow cytometry, supporting their utility as robust indicators of early radiation-induced hematopoietic injury.

### 3.5. Radiosensitive HSPC Subsets Remain Detectable After Short-Term In Vitro Culture

**Figure 5:**
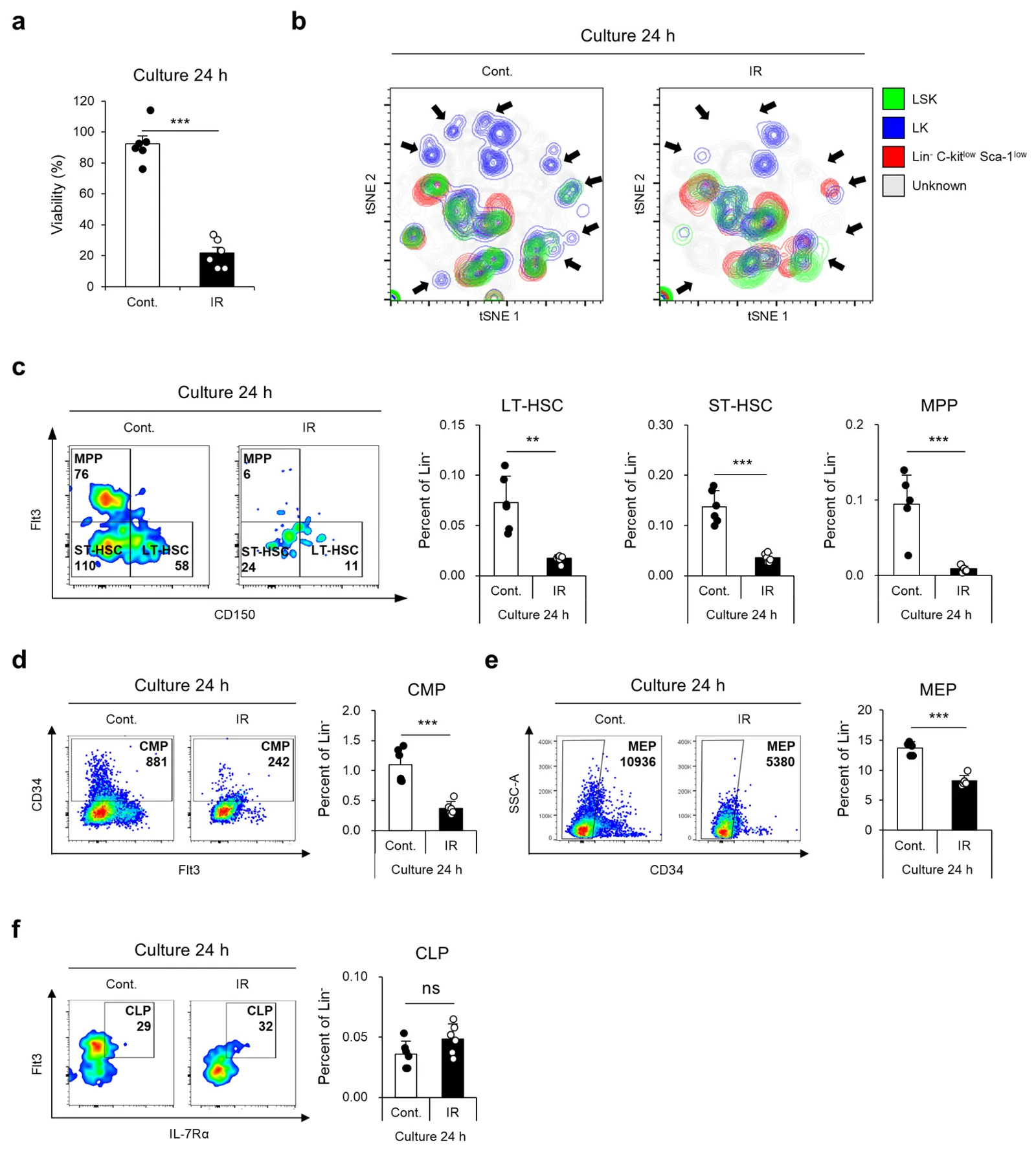
Cultured BM cells show a different pattern of LT-HSC, CMP, and MEP compared to primary BM cells. BM cells isolated from irradiated mice were cultured in 96-well plates (5 × 10^5^ or 2 × 10^6^ cells) for 24 h. (**a**) Cell viability was measured using CCK-8. The 24 h survival rate was evaluated based on 0 h. (**b**) The populations in LSK (Lin^−^ Sca-1^+^ c-Kit^+^), LK (Lin^−^ Sca-1^−^ c-Kit^+^), and Lin^−^ Sca-1^low^ c-Kit^low^ among Lin^−^ cells were visualized using a t-SNE map. The black arrows indicate areas on the tSNE map where there is a large difference between groups. (**c**–**f**) After performing Lin^−^ (anti-CD3ε, anti-Gr-1, anti-CD11c, anti-CD11b, anti-CD19, and anti-NK1.1) gating, only HSPCs (c-Kit, Sca-1) remained and were then gated individually in combination to identify each subpopulation. (**c**) After performing c-Kit^+^ and Sca-1^+^ gating, LT-HSC (Flt3^−^ CD150^+^), ST-HSC (Flt3^−^ CD150^−^), and MPP (Flt3^+^ CD150^−^) were identified using Flt3 and CD150. (**d**) c-Kit^+^, Sca-l^−^, CD34^+^, and Flt3^+/−^ were identified CMP. (**e**) c-Kit^+^, Sca-l^−^, and CD34^+^ were identified MEP. (**f**) c-Kit^low^, Sca-l^low^, IL-7Rα^+^, and Flt3^+^ was identified CLP. The HSPCs population was quantified through flow cytometry. The graph shows the percentage (%) of the LT-HSC, ST-HSC, MPP, CMP, MEP, and CLP. Dot plots presented representative images and represented the mean cell number values. Data were presented as mean ± SD. The reported *p*-values were obtained from Student’s *t*-tests. (** *p* < 0.01, *** *p* < 0.001, ns: not significant).

To evaluate whether radiation-induced alterations in HSPC populations could be monitored under in vitro screening conditions, bone marrow cells isolated from irradiated mice were cultured for 24 h prior to analysis. Following culture, cell viability in the irradiated group decreased to approximately 22% (Figure 5a). Consistent with this reduction, irradiated group cells exhibited abnormal morphology characterized by irregular and shrunken cellular structures (Figure S3a). Although total bone marrow cell numbers were not significantly different between irradiated and non-irradiated groups (Figure S3b), the proportion of live cells was significantly reduced following irradiation (Figure S3c).

Flow cytometric analysis demonstrated a marked reduction in the LK population after irradiation, whereas LSK and Lin^−^ Sca-1^low^ c-Kit^low^ populations remained relatively unchanged (Figure 5b). To determine whether radiosensitive HSPC subsets remained detectable after culture, six major HSPC populations were analyzed using the same gating strategy applied at 0 h (Figure S3d). LT-HSCs, ST-HSCs, and MPPs were significantly reduced by 76%, 74%, and 91%, respectively, following irradiation (Figure 5c). Similarly, MEPs exhibited a significant reduction of approximately 40% (Figure 5e). In contrast, CMPs and CLPs showed no significant changes after irradiation (Figure 5d,f). To further evaluate temporal changes during culture, HSPC populations were compared between 0 h and 24 h. LT-HSCs remained stable in non-irradiated controls but exhibited an additional 71% reduction in irradiated cultures (Figure S3e). ST-HSC frequencies remained relatively unchanged during culture regardless of irradiation status (Figure S3f). MPPs declined during culture even in non-irradiated controls, eventually reaching levels similar to those observed in irradiated samples at 0 h (Figure S3g). CMPs increased during culture in control samples (Figure S3h), whereas MEPs and CLPs exhibited only minor changes (Figure S3i,j).

Collectively, these findings demonstrate that radiation-induced alterations in ST-HSCs, MPPs, and MEPs remain detectable following short-term culture, supporting their suitability for monitoring hematopoietic injury under in vitro screening conditions.

## 4. Discussion

The development of effective medical countermeasures for H-ARS remains an important global priority because currently approved therapies provide only partial protection and are often difficult to implement during large-scale radiation emergencies [25,26,27,28]. Although substantial progress has been made in understanding radiation-induced DNA damage and hematopoietic failure, practical cellular endpoints that can be used for rapid therapeutic screening remain poorly defined. In the present study, we combined high-dimensional CyTOF profiling with conventional flow cytometry to identify radiosensitive HSPC populations and establish biologically relevant endpoints for H-ARS therapeutic screening.

A major finding of this study is that early radiation-induced hematopoietic injury is characterized by selective remodeling of HSPC subsets rather than uniform depletion of the entire stem-cell compartment. CyTOF analysis revealed that total HSPCs rapidly declined following 6.5 Gy irradiation, decreasing by approximately 50% during the early phase and nearly 90% by 24 h. However, this reduction was not equally distributed among HSPC populations. LT-HSCs were relatively preserved, whereas ST-HSCs, MPPs, and MEPs exhibited marked reductions. These findings are consistent with previous studies demonstrating that quiescent LT-HSCs possess enhanced resistance to radiation-induced stress through reduced proliferative activity and superior DNA repair capacity [29,30,31,32]. In contrast, actively proliferating progenitor populations are more susceptible to radiation-induced damage, leading to preferential depletion during the earliest stages of hematopoietic collapse [33]. In addition, bone marrow niche- and metabolism-related mechanisms may influence HSPC radiosensitivity: megakaryocytes support HSC quiescence and post-injury regeneration [34,35], megakaryocyte-derived IGF1 limits radiation-induced HSC ferroptosis [36], and cholesterol-dependent ferroptosis resistance and mitochondrial serine catabolism promote HSC maintenance and hematopoietic recovery under irradiation-induced stress [37]. Taken together, these observations suggest that radiation injury follows a hierarchical pattern of HSPC vulnerability, with specific radiosensitive progenitor populations serving as biologically informative indicators of early hematopoietic dysfunction.

Another important observation was the identification of a narrow temporal window during which radiation-induced hematopoietic remodeling can be effectively monitored. Although cell viability was already reduced to less than 25% shortly after irradiation, surviving bone marrow cells retained partial differentiation capacity in response to GM-CSF stimulation. By 24 h post-irradiation, however, both cellularity and functional potential were substantially diminished. These findings suggest that the earliest phase following radiation exposure may represent the most informative period for evaluating candidate radiomitigators. While many previous studies have focused on later endpoints such as survival, peripheral blood recovery, or bone marrow regeneration [6,38,39,40], our results indicate that critical biological changes occur within the first several hours after exposure. An important implication of our findings is that early post-irradiation time points represent a therapeutic window rather than simply an earlier stage of inevitable cell loss. Although some HSPCs that survive initially may subsequently lose viability or function, the early phase captures cells that have sustained sublethal damage and therefore remain potentially recoverable through timely intervention. Consequently, early alterations in radiosensitive HSPC subsets provide biologically meaningful pharmacodynamic endpoints for evaluating candidate radioprotective or radiomitigating agents before irreversible hematopoietic collapse occurs. This concept may improve the sensitivity of preclinical therapeutic screening by enabling efficacy assessment at a stage when hematopoietic recovery remains achievable. Therefore, early assessment of radiosensitive HSPC populations may provide a more sensitive and quantitative measure of therapeutic efficacy. Although radiation exposure may influence the expression of individual surface markers, our findings are supported by consistent results from both CyTOF and conventional flow cytometry, together with marked reductions in bone marrow cellularity and cell viability, indicating that the observed changes primarily reflect genuine hematopoietic injury rather than surface marker modulation alone. While immunophenotypic profiling does not fully define long-term HSPC function, it provides biologically relevant endpoints for early therapeutic screening. Future studies incorporating competitive repopulation and serial transplantation assays will further establish the relationship between these early phenotypic changes and long-term hematopoietic recovery.

A distinguishing feature of this study is the translation of high-dimensional CyTOF discoveries into a simplified flow cytometry-based screening strategy. CyTOF enabled unbiased characterization of radiation-induced remodeling across multiple HSPC populations, whereas flow cytometry provided a practical platform for routine evaluation. Importantly, ST-HSCs, MPPs, and MEPs consistently exhibited robust reductions across both analytical platforms and remained detectable under short-term culture conditions. This reproducibility suggests that these populations represent reliable cellular endpoints for therapeutic screening. Unlike conventional viability-based assays, which provide limited information regarding hematopoietic hierarchy, monitoring specific radiosensitive HSPC subsets offers direct insight into the preservation of regenerative capacity within the bone marrow compartment.

Current approaches for evaluating H-ARS countermeasures primarily rely on animal survival, hematologic recovery, bone marrow cellularity, or general measures of cell viability [18,19,20]. Although these endpoints remain essential for regulatory approval and efficacy validation, they often require prolonged observation periods and provide limited resolution regarding early hematopoietic injury. In contrast, the framework proposed in this study focuses on quantifiable cellular populations that respond rapidly and reproducibly to radiation exposure. By integrating CyTOF-based discovery with flow cytometry-based validation, our approach provides a practical strategy for identifying candidate compounds capable of preserving radiosensitive HSPC populations during the earliest phase of injury.

## 5. Conclusions

The choice of 6.5 Gy was intended to establish a biologically informative balance between injury severity and therapeutic recoverability. While lower doses produce limited hematopoietic damage and higher doses cause extensive irreversible cell loss, 6.5 Gy generates substantial depletion of radiosensitive HSPCs while preserving a residual population of damaged but potentially recoverable cells. This creates a clinically relevant therapeutic window in which candidate radioprotective or radiomitigating agents can be evaluated based on their ability to preserve hematopoietic integrity before irreversible bone marrow failure occurs [41]. Using high-dimensional CyTOF analysis, we identified radiosensitive progenitor populations that undergo rapid changes following irradiation and subsequently translated these findings into a simplified flow cytometry-based platform. Among the analyzed populations, ST-HSCs, MPPs, and MEPs consistently exhibited robust and reproducible responses to radiation exposure, highlighting their potential as practical cellular endpoints for H-ARS therapeutic screening. Importantly, these changes emerged during the earliest phase of hematopoietic injury, suggesting that this time window may provide a sensitive opportunity for evaluating candidate radiomitigators. Collectively, our findings establish an early hematopoietic injury framework that links high-dimensional immune profiling with practical screening applications and provide a biologically informed strategy for future H-ARS therapeutic discovery and evaluation.

## Figures and Tables

**Table 1:** List of antibodies for the 19 panels.

Specificity	Clone	Metal Isotype	Purpose
CD45	30-F11	89Y	Pan leukocytes
Ly6G/c [Gr1]	RB6-8C5	141pr	Granulocytes lineage
CD11c	N418	142Nd	Dendritic cells lineage
CD115(CSF-1R)	AFS98	144Nd	Monocytes progenitors
CD11b [MAC1]	M1/70	148Nd	Macrophages lineage
CD19	6D5	149Sm	B cells lineage
Ly-6C	HK1.4	150Nd	Monocyte
CD3e	145-2C11	152Sm	T cells lineage
CD16/32	93	153Eu	Myeloid progenitors
TER-119	TER-119	154Sm	Erythroid cells lineage
CD172a	P84	155Gd	pre-cDC
CD45R(B220)	RA3-6B2	160Gd	B cells lineage
Ly6A/E(Sca-1)	D7	164Dy	Stem cells
CD161/NK 1.1	PK136	165Ho	NK cells lineage
CD117(c-kit)	2B8	166Er	Stem/progenitors
CD150	TC15-12F12.2	167Er	LT-HSC
CD135(Flt3)	A2F10	173Yb	Progenitor marker
CD127(IL-7Ra)	A7R34	174Yb	Lymphoid progenitors
CD34	MEC14.7	176Yb	Progenitor marker

## Data Availability

The original contributions presented in this study are included in the article/Supplementary Materials. Further inquiries can be directed to the corresponding author.

## Supplementary Materials

The following supporting information can be downloaded at https://www.mdpi.com/article/10.3390/cimb48080801/s1.

## Abbreviations

The following abbreviations are used in this manuscript:

H-ARS	Hematopoietic Acute Radiation Syndrome
GI-ARS	Gastrointestinal Acute Radiation Syndrome
CNS-ARS	Cerebrovascular Acute Radiation Syndrome
BM	Bone Marrow
DEARE	Delayed Effects of Acute Radiation Exposure
DSB	Double-Strand Break
HSPC	Hematopoietic Stem and Progenitor Cell
LT-HSC	Long-Term Hematopoietic Stem Cell
ST-HSC	Short-Term Hematopoietic Stem Cell
MPP	Multipotent Progenitor
MEP	Megakaryocyte–Erythroid Progenitor
CMP	Common Myeloid Progenitor
CLP	Common Lymphoid Progenitor
GMP	Granulocyte–Monocyte Progenitor
MDP	Monocyte–Dendritic cell Progenitor
CDP	Common Dendritic cell Progenitor
MCM	Medical Countermeasure
G-CSF	Granulocyte Colony-Stimulating Factor
GM-CSF	Granulocyte–Macrophage Colony-Stimulating Factor
CyTOF	Cytometry by Time-Of-Flight
t-SNE	t-distributed Stochastic Neighbor Embedding
Opt-SNE	Optimized t-distributed Stochastic Neighbor Embedding
FlowSOM	Flow Cytometry using Self-Organizing Maps
TBI	Total-Body Irradiation

## References

[1] Bernier J., Hall E., Giaccia A. (2004). Radiation oncology: A century of achievements. Nat. Rev. Cancer.

[2] Huynh E., Hosny A., Guthier C., Bitterman D.S., Petit S.E., Haas-Kogan D.A., Kann B., Aerts H.J.W.L., Mak R.H. (2020). Artificial intelligence in radiation oncology. Nat. Rev. Clin. Oncol..

[3] Henry E., Arcangeli M.L. (2021). How hematopoietic stem cells respond to irradiation: Similarities and differences between low and high doses of ionizing radiations. Exp. Hematol..

[4] Freeman M.L. (2025). Gastrointestinal acute radiation syndrome: Current knowledge and perspectives. Cell Death Discov..

[5] Christy B.A., Herzig M.C., Wu X., Mohammadipoor A., McDaniel J.S., Bynum J.A. (2024). Cell therapies for acute radiation syndrome. Int. J. Mol. Sci..

[6] Vercellino J., Małachowska B., Kulkarni S., Bell B.I., Shajahan S., Shinoda K., Eichenbaum G., Verma A.K., Ghosh S.P., Yang W.L. (2024). Thrombopoietin mimetic stimulates bone marrow vascular and stromal niches to mitigate acute radiation syndrome. Stem Cell Res. Ther..

[7] Venkateswaran K., Shrivastava A., Agrawala P.K., Prasad A.K., Kalra N., Pandey P.R., Manda K., Raj H.G., Parmar V.S., Dwarakanath B.S. (2016). Mitigation of radiation-induced hematopoietic injury by the polyphenolic acetate 7,8-diacetoxy-4-methylthiocoumarin in mice. Sci. Rep..

[8] Sharma N.K., Holmes-Hampton G.P., Kumar V.P., Biswas S., Wuddie K., Stone S., Aschenake Z., Wilkins W.L., Fam C.M., Cox G.N. (2020). Delayed effects of acute whole body lethal radiation exposure in mice pre-treated with BBT-059. Sci. Rep..

[9] Singh V.K., Wise S.Y., Fatanmi O.O., Petrus S.A., Carpenter A.D., Lugo-Roman L.A., Lee S.H., Hauer-Jensen M., Seed T.M. (2024). Pathology of acute sub-lethal or near-lethal irradiation of nonhuman primates prophylaxed with the nutraceutical, gamma tocotrienol. Sci. Rep..

[10] Bigildeev A., Bigildeev E., Bulygina E., Tsygankova S., Gusakova M., Illarionova O. (2025). Preliminary high-dose irradiation of the recipient and associated damage of bone marrow stromal compartment enables bone marrow stroma transplantation. Sci. Rep..

[11] Guo C.Y., Luo L., Urata Y., Goto S., Huang W.J., Takamura S., Hayashi F., Doi H., Kitajima Y., Ono Y. (2015). Sensitivity and dose dependency of radiation-induced injury in hematopoietic stem/progenitor cells in mice. Sci. Rep..

[12] Biechonski S., Olender L., Zipin-Roitman A., Yassin M., Aqaqe N., Marcu-Malina V., Rall-Scharpf M., Trottier M., Meyn M.S., Wiesmüller L. (2018). Attenuated DNA damage responses and increased apoptosis characterize human hematopoietic stem cells exposed to irradiation. Sci. Rep..

[13] Fang H., Xie X., Liu P., Rao Y., Cui Y., Yang S., Yu J., Luo Y., Feng Y. (2020). Ziyuglycoside II alleviates cyclophosphamide-induced leukopenia in mice via regulation of HSPC proliferation and differentiation. Biomed. Pharmacother..

[14] Bruno F., Georgiou C., Cunningham D., Bett L., Secchi M.A., Atkinson S., Antón S.G., Birch F., Langhorne J., Celso C.L. (2025). Differential response and recovery dynamics of HSPC populations following *Plasmodium chabaudi* infection. Int. J. Mol. Sci..

[15] Tang X., Wang Z., Wang J., Cui S., Xu R., Wang Y. (2023). Functions and regulatory mechanisms of resting hematopoietic stem cells: A promising targeted therapeutic strategy. Stem Cell Res. Ther..

[16] Zhang Q., Rydström A., Hidalgo I., Cammenga J., Nilsson A.R. (2025). Medium-dose irradiation impairs long-term hematopoietic stem cell functionality and hematopoietic resilience to cytotoxic stress. Int. J. Biochem. Cell Biol..

[17] Zhang Y., Chen X., Wang X., Chen J., Du C., Wang J., Liao W. (2024). Insights into ionizing radiation-induced bone marrow hematopoietic stem cell injury. Stem Cell Res. Ther..

[18] Kiang J.G., Cannon G., Singh V.K. (2024). An overview of radiation countermeasure development in Radiation Research from 1954 to 2024. Radiat. Res..

[19] Farese A.M., Cohen M.V., Stead R.B., Jackson W., Macvittie T.J. (2012). Pegfilgrastim administered in an abbreviated schedule significantly improved neutrophil recovery after high-dose radiation-induced myelosuppression in rhesus macaques. Radiat. Res..

[20] DiCarlo A.L., Horta Z.P., Aldrich J.T., Jakubowski A.A., Skinner W.K., Case C.M. (2019). Use of growth factors and other cytokines for treatment of injuries during a radiation public health emergency. Radiat. Res..

[21] Horta Z.P., Case C.M., DiCarlo A.L. (2019). Use of growth factors and cytokines to treat injuries resulting from a radiation public health emergency. Radiat. Res..

[22] Benderitter M., Herrera-Reyes E., Gigov Y., Souleau B., Huet C., Trompier F., Fagot T., Gregoire E., Malfuson J.V., Konopacki-Potet J. (2021). Hematopoietic recovery using multi-cytokine therapy in 8 patients presenting radiation-induced myelosuppression after radiological accidents. Radiat. Res..

[23] Sim H.B., Choi Y.J., Mun S.K., Ramos S.C., Park D.H., Lee J.B., Jeong S.H., Kim J.J. (2025). Analysis of immune cell remodeling and functional alterations induced by aging and obesity in mice. Int. Immunopharmacol..

[24] Sim H.B., Jang J.H., Mun S.K., Ramos S.C., Park D.H., Choi Y.J., Han J.Y., Lee J.B., Chung H.S., Lee K.B. (2026). Reading the immune clock: A machine learning model predicts mouse immune age from cellular patterns. Nat. Commun..

[25] Wesolowski R., Fish B.L., Eibl M., Bähr S., Mehta S.M., Czajkowski M.T., Gasperetti T., Orschell C.M., Asang C., Singh N. (2025). IEPA, a novel radiation countermeasure, alleviates acute radiation syndrome in rodents. Int. J. Radiat. Biol..

[26] Qian L., Cen J. (2020). Hematopoietic stem cells and mesenchymal stromal cells in acute radiation syndrome. Oxidative Med. Cell. Longev..

[27] Micewicz E.D., Kim K., Iwamoto K.S., Ratikan J.A., Cheng G., Boxx G.M., Damoiseaux R.D., Whitelegge J.P., Ruchala P., Nguyen C. (2017). 4-(Nitrophenylsulfonyl)piperazines mitigate radiation damage to multiple tissues. PLoS ONE.

[28] Kim K., Pollard J.M., Norris A.J., McDonald J.T., Sun Y., Micewicz E., Pettijohn K., Damoiseaux R., Iwamoto K.S., Sayre J.W. (2009). High-throughput screening identifies two classes of antibiotics as radioprotectors: Tetracyclines and fluoroquinolones. Clin. Cancer Res..

[29] Mohrin M., Bourke E., Alexander D., Warr M.R., Barry-Holson K., Le Beau M.M., Morrison C.G., Passegué E. (2010). Hematopoietic stem cell quiescence promotes error-prone DNA repair and mutagenesis. Cell Stem Cell.

[30] Suda T., Takubo K., Semenza G.L. (2011). Metabolic regulation of hematopoietic stem cells in the hypoxic niche. Cell Stem Cell.

[31] Milyavsky M., Gan O.I., Trottier M., Komosa M., Tabach O., Notta F., Lechman E., Hermans K.G., Eppert K., Konovalova Z. (2010). A distinctive DNA damage response in human hematopoietic stem cells reveals an apoptosis-independent role for p53 in self-renewal. Cell Stem Cell.

[32] Li X., Sipple J., Pang Q., Du K.W. (2012). Salidroside stimulates DNA repair enzyme Parp-1 activity in mouse HSC maintenance. Blood.

[33] Pajonk F., Vlashi E., McBride W.H. (2010). Radiation resistance of cancer stem cells: The 4 R’s of radiobiology revisited. Stem Cells.

[34] Zhao M., Perry J.M., Marshall H., Venkatraman A., Qian P., He X.C., Ahamed J., Li L. (2014). Megakaryocytes maintain homeostatic quiescence and promote post-injury regeneration of hematopoietic stem cells. Nat. Med..

[35] Bruns I., Lucas D., Pinho S., Ahmed J., Lambert M.P., Kunisaki Y., Scheiermann C., Schiff L., Poncz M., Bergman A. (2014). Megakaryocytes regulate hematopoietic stem cell quiescence through CXCL4 secretion. Nat. Med..

[36] Liao W., Chen X., Zhang S., Chen J., Liu C., Yu K., Zhang Y., Chen M., Chen F., Shen M. (2024). Megakaryocytic IGF1 coordinates activation and ferroptosis to safeguard hematopoietic stem cell regeneration after radiation injury. Cell Commun. Signal..

[37] Du C., Liu C., Yu K., Zhang S., Fu C., Liao W., Chen J., Zhang Y., Wang X., Chen M. (2024). Mitochondrial serine catabolism safeguards maintenance of the hematopoietic stem cell pool in homeostasis and injury. Cell Stem Cell.

[38] Diaz M.F., Horton P.D., Dumbali S.P., Kumar A., Livingston M., Skibber M.A., Mohammadalipour A., Gill B.S., Zhang S., Cox C.S. (2020). Bone marrow stromal cell therapy improves survival after radiation injury but does not restore endogenous hematopoiesis. Sci. Rep..

[39] Farese A.M., Booth C., Tudor G.L., Cui W., Cohen E.P., Parker G.A., Hankey K.G., MacVittie T.J., Thomas J. (2021). The natural history of acute radiation-induced H-ARS and concomitant multi-organ injury in the non-human primate: The MCART experience. Health Phys..

[40] Micewicz E.D., Iwamoto K.S., Ratikan J.A., Nguyen C., Xie M.W., Cheng G., Boxx G.M., Deriu E., Damoiseaux R.D., Whitelegge J.P. (2019). The aftermath of surviving acute radiation hematopoietic syndrome and its mitigation. Radiat. Res..

[41] Orschell C.M., Wu T., Patterson A.M. (2022). Impact of Age, Sex, and Genetic Diversity in Murine Models of the Hematopoietic Acute Radiation Syndrome (H-ARS) and the Delayed Effects of Acute Radiation Exposure (DEARE). Curr. Stem Cell Rep..

