# Supplementary material for: Radiosensitive HSPC Subsets Define Early Hematopoietic Injury and Enable H-ARS Therapeutic Screening
Source: Curr Issues Mol Biol. 2026 Aug 7;48(8):801. doi: 10.3390/cimb48080801 (PMC13510691; doi:10.3390/cimb48080801)
Supplement: Supplementary file 1 [file cimb-48-00801-s001.zip › cimb-4450832-supplementary.pdf]

Supplementary Materials

Radiosensitive HSPC Subsets Define Early Hematopoietic Injury and Enable H-ARS Therapeutic Screening

Hyun Bo Sim [1,†], Dae-Han Park [1,†], Seul-Ki Mun [1,2], Yu-Jeong Choi [1,3], Ho Seong Seo [4], Seung-Hyun Jeong [5] , Dong-Jo Chang [5,*] and Jong-Jin Kim [1,*]

[1] Department of Biomedical Science, Sunchon National University, 255 Jungang-ro, Suncheon-si 57922, Republic of Korea; kokonun3@naver.com (H.B.S.); carbdsinc@naver.com (D.-H.P.); motomoto1210@naver.com (S.-K.M.); pskd357@naver.com (Y.-J.C.)

[2] R&D Team, Suncheon Research Center for Bio Health Care, Suncheon, 57962, Republic of Korea

[3] Korea Radio-Isotope Center for Pharmaceuticals, Korea Institute of Radiological & Medical Sciences, Seoul 01812, Republic of Korea

[4] Research Division for Cyclotron Application, Korea Atomic Energy Research Institute, Jeongeup 56212, Republic of Korea; hoseongseo@kaeri.re.kr

[5] Research Institute of Life and Pharmaceutical Sciences, College of Pharmacy, Sunchon National University, Suncheon-si 57922, Republic of Korea; jeongsh@scnu.ac.kr

* Correspondence: djchang@scnu.ac.kr (D.-J.C.); kimjj@scnu.ac.kr (J.-J.K.)

† These authors contributed equally to this work.

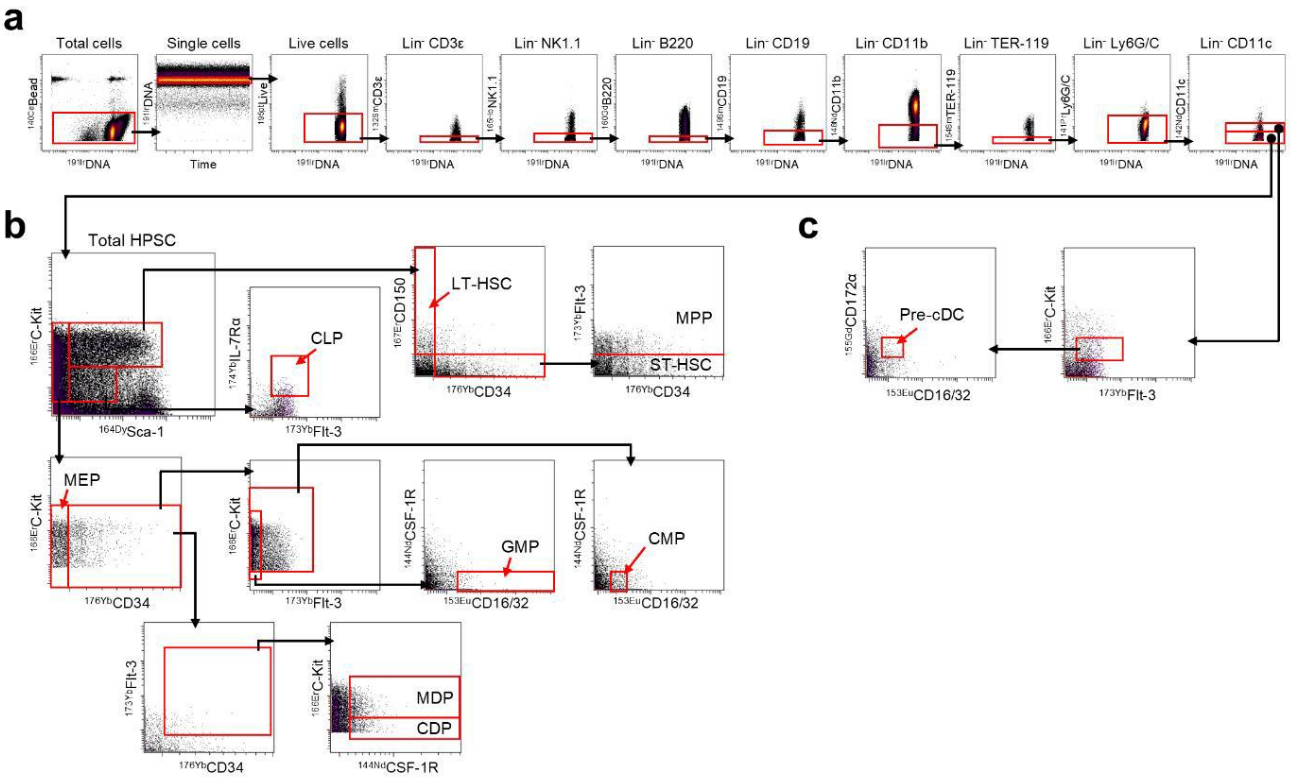

Figure S1. CyTOF-based identification of HSPC population changes. (**a**) Lineage-out gating strategy to remove fully differentiated cells that are not HSPCs, leaving only single cells. (**b**) Based on the Lin$^-$ event, the Sca-1$^+$ c-Kit$^+$(LSK) population was selected, and LSK CD150$^+$ CD34$^-$ was named LT-HSC, LSK CD150$^-$ CD34$^+$ Flt-3$^-$ was named ST-HSC, and LSK CD150$^-$ CD34$^+$ Flt-3$^+$ was named MPP. Lin$^-$ Sca-1^{low} c-Kit^{low} Flt-3$^+$ IL-7Rα^+ was named CLP. Lin$^-$ Sca-1- c-Kit$^+$(LK) population was selected, and LK CD34$^-$ was named MEP, LK^{low} CD34$^+$ Flt-3$^+$ CSF-1R$^+$ was named CDP, LK^{high} CD34$^+$ Flt-3$^+$ CSF-1R$^+$ was named MDP, LK Flt-3$^-$ CSF-1R$^-$ CD16/32^{high} was named GMP, and LK Flt-3$^{+/-}$ CSF-1R$^-$ CD16/32^{low} was named CMP. (**c**) At the end of the lineage out process, the CD11c^{low} population was selected instead of the CD11c population, and c-Kit^{low} Flt-9 3$^+$ CD16/32^{low} CD172α^{low} was named Pre-cDC.

a

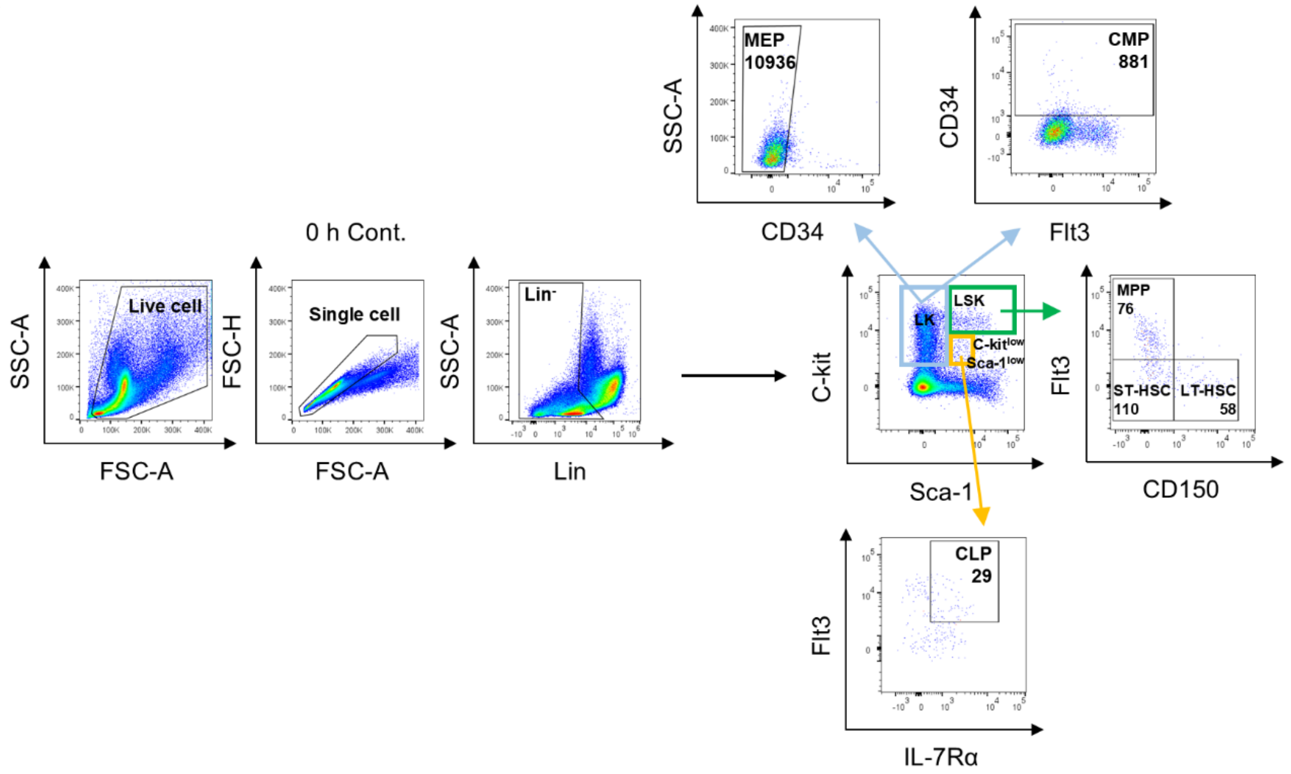

Figure S2. Flow cytometry gate for HSPCs analysis. (a) Gate storage for analysis of HSPCs in culture 0 h was shown. Dot plots present representative images and represent the mean cell number values.

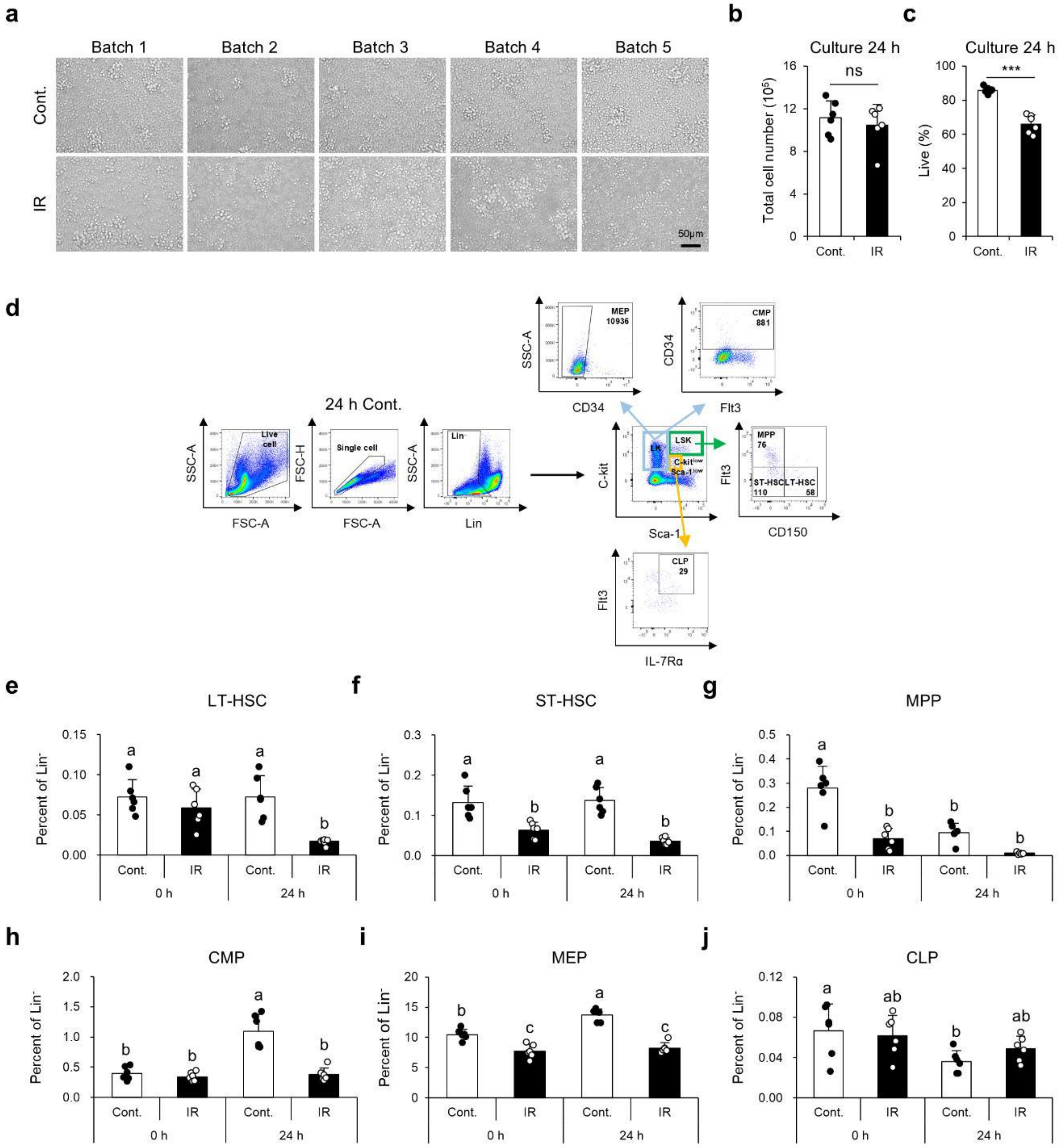

Figure S3. Changes in BM cells cultured for 24 h. Irradiated BM cells were cultured in 96-well plates (2×10^6 cells) for 24 h. (a) BM cells were imaged using an M7000 microscope (×10 air objective). (b, c) Total cell number and live (%) of BM cells were measured using a cell counter. (d) Gate storage for analysis of HSPCs in culture for 24 h is shown. (e-j) The culture 0 h and culture 24 h were combined with the HSPCs data. Dot plots presented representative images and represent the mean cell number values. Data are presented as mean ± SD. The reported *p*-values were obtained from a one-way analysis of variance (ANOVA). Different letters (a-c) indicate significant differences.
